# TAPPR PCR assay design—targeted, automated, primer and probe retrieval for scalable molecular assay design

**DOI:** 10.1093/bioadv/vbag274

**Published:** 2026-09-17

**Authors:** Phillip E Davis, Colin W Price, Taylor Otwell, Anshika Kapoor, Vita Domnenko, Joseph A Russell

**Affiliations:** Integrated Health Surveillance and Diagnostics of MRIGlobal, Kansas City, MO 64110, United States; Integrated Health Surveillance and Diagnostics of MRIGlobal, Kansas City, MO 64110, United States; Integrated Health Surveillance and Diagnostics of MRIGlobal, Kansas City, MO 64110, United States; Integrated Health Surveillance and Diagnostics of MRIGlobal, Kansas City, MO 64110, United States; Integrated Health Surveillance and Diagnostics of MRIGlobal, Kansas City, MO 64110, United States; Integrated Health Surveillance and Diagnostics of MRIGlobal, Kansas City, MO 64110, United States

## Abstract

**Motivation:**

The rapid and reliable detection of infectious disease agents is critical for effective biosurveillance, diagnostics, and outbreak response. However, existing molecular assay design methods face significant limitations in scalability, speed, and adaptability to rapidly evolving pathogens, often leading to outdated or suboptimal assays. Here, we present Targeted, Automated Primer and Probe Retrieval (TAPPR), a novel, automated pipeline for scalable molecular assay design. TAPPR uses alignment-free methods to identify conserved regions and marker sequences across large-scale genomic datasets, supporting customizable design parameters for inclusivity, exclusivity, and assay specificity. To evaluate TAPPR’s performance, assays were designed for diverse microbial targets, including Mpox, *Mycobacterium tuberculosis*, SARS-CoV-2, and *Candida albicans*, representing viral, bacterial, and fungal pathogens. The pipeline incorporates k-mer set operations and clustering strategies to address sequence diversity and streamline conserved region identification. Designed assays underwent in silico PCR simulations and laboratory testing to assess specificity, sensitivity, and exclusivity. Results demonstrated high accuracy across targets, with superior sensitivity to existing fielded assays where available. Additionally, we compare TAPPR to alternative available molecular assay design tools to demonstrate its advantages. This work highlights TAPPR’s capability to accelerate the development of molecular diagnostics by efficiently leveraging vast genomic datasets and addressing computational bottlenecks. TAPPR represents a scalable, adaptable tool for rapidly designing high-quality molecular assays, positioning itself as a critical asset for biosurveillance and public health response to emerging and re-emerging infectious disease threats.

**Results:**

TAPPR demonstrates rapid and scalable data-driven molecular assay design through alignment-free estimations of conserved regions and marker regions. Through both *in silico* and lab bench evaluation, TAPPR assays are demonstrated to perform equivalently or better than previously utilized publicly available qPCR assays for emergent disease diagnostics. TAPPR is also shown to produce results where other available automated molecular assays design solutions fail to do so on the order of hours.

**Availability and implementation:**

TAPPR is available at https://github.com/mriglobal/tappr under a Creative Commons Attribution-NonCommercial-NoDerivatives 4.0 International Public License.

## 1 Introduction

Molecular diagnostics have steadily become an indispensable tool in monitoring infectious disease and the clinical diagnosis of disease, culminating with the COVID-19 pandemic where more than 500 different SARS-CoV-2 PCR assays became commercially available (Linkowska *et al*. 2023). Organizations charged with promoting human health such as the World Health Organization (WHO) and Center for Disease Control (CDC) and animal health, such as the World Organization for Animal Health (WOAH) have relied on molecular technologies for the characterization of various threats to human health, such as *Mycobacterium tuberculosis* drug resistance (Leung *et al*. 2022), diagnosis of Mpox in human patients in the 2022 and 2024 outbreaks (da Silva *et al*. 2023, WHO 2024), and monitoring the spread of highly pathogenic H5N1 in livestock in North America in 2024 (Sahoo *et al*. 2024). Since the advent of PCR, many different diagnostic approaches have used the technology for the purposes of nucleic acid detection or characterization. Example methods include real-time quantitative PCR using an intercalating agent, probe-based PCR that relies on the integration of an internal oligonucleotide, and amplicon sequencing, where sequencing the content of the amplicon provides the diagnostic utility.

Despite its prominent role in public and animal health, there are many areas where the development and maintenance of these molecular diagnostics can be improved. Assay design is typically on the order of weeks or months, target selection is often constrained by research interest in specific genes, and there are computational bottlenecks in traditional methods that make the utilization of expanding sequence data difficult (SantaLucia *et al*. 2020).

A consequence of these limitations has been several preventable issues in molecular assays being used for these purposes. In 2018, African Swine Fever Virus (ASFV), which is a double-stranded DNA virus with a nearly 100% mortality rate in domestic pigs, was spreading rapidly through Asia causing large economic losses for countries in the region (You *et al*. 2021). The WOAH issued a field guide for molecular detection of ASFV. However, the molecular assay used was designed initially in 2003 (King *et al*. 2003) and in the 15 years between that initial design and the 2018 outbreak, GenBank sequences published for ASFV had already shown mismatches accumulating in the p72 gene targeted by the assay. A mismatch in the probe sequence resulted in PCR false negatives (Truong *et al*. 2020). Similarly, in the 2022 Mpox outbreak, the CDC generic molecular protocol for Mpox detection that was issued in June of 2022, used an assay designed in 2010 (Li *et al*. 2010). The design rationale made by the authors targeted the TNF receptor gene because it was a site of common deletions among Orthopox clades. Unsurprisingly, this led to a failure of the assay to detect Mpox cases in September 2022, when a deletion occurred in the target region of the TNF receptor. This was foreseeable, not just from the original author’s rationale for target selection, but as of July 2022, the primers and probes for the CDC generic Mpox assay only had perfect matches for 38 of the 402 available complete Mpox genomes on NCBI Virus (NCBI Virus 2004). Empirically, the evidence for the degradation of the assay’s performance was already available.

This effort seeks to remediate these issues by making design and evaluation of molecular assays for targeted detection both simplified and scalable, with the goal of improving the speed and quality of response from human and animal health organizations to infectious disease outbreaks. Here, we present Targeted Primer and Probe Retrieval (TAPPR), an automated molecular assay design pipeline that utilizes alignment-free approaches for the identification of molecular marker and conserved regions with an oligonucleotide selection algorithm based on the assay parameters specified by the user. The focus on scalability by using alignment-free methods unlocks advantages in terms of time-to-answer, but also in utilizing much larger amounts of genomic data input in the search for candidate assays both in terms of the number of genome representatives and the sizes of these genomes. We choose several representative target clades and show how our design pipeline can be configured to produce candidate assays, as well as wet lab data showing their performance against other, currently fielded examples. The target pathogens selected were Mpox, *M. tuberculosis*, SARS-CoV-2, and *Candida albicans*. These targets represent diverse genomic architectures and clinical contexts—including DNA and RNA viruses, bacterial pathogens with high genomic plasticity, and complex eukaryotic organisms—demonstrating TAPPR’s flexibility across a broad spectrum of diagnostic challenges.

The development of new tools in automated molecular assay design is ongoing. We attempt to compare TAPPR to other available tools with similar design goals, both alignment-based, such as DECIPHER (Wright 2016), and alignment-free, such as Rapid Identification of PCR Primer Pairs for Unique Sequences (RUCS) (Thomsen *et al*. 2017) and demonstrate how TAPPR improves over these existing methods.

## 2 Methods

### 2.1 Data

For the design of the assays presented, we used either NCBI Datasets (Cox *et al*. 2025) or NCBI Virus to gather the sequence and meta data.

For design of all primers and probes, the complete set of inclusive sequences in Table 1 were used to find regions of conservation, except for SARS-CoV-2. Since the number of sequences was so large (*N* = 1 011 430) and estimated conserved regions require k-mers to be inclusive to all input records, even a small amount of random sequencing error distributed uniformly across the genome sequences could result in 0 estimated conserved regions. Therefore, in the case of SARS-CoV-2, we randomly subsampled 2000 sequences, stratified by the Pangolin-assigned (O’Toole et al. 2021) lineage provided in the NCBI Virus metadata, for the design of primers and probes and evaluated inclusivity against the entire dataset. To estimate the effectiveness of this strategy we performed this random subsampling and evaluation procedure 10 times to find a mean and standard deviation inclusivity statistics for this method.

**Table 1:** Sequence data used in design and evaluation.^a^

Target	Taxid	No. of inclusive sequences	No. of exclusive sequences	Date accessed
**Mpox (both Clade I and Clade II)**	10244	402	2075	27 July 2022
**SARS-CoV-2**	2697049	1 011 430	N/A	16 June 2023
***M. tuberculosis* complex**	77643	58	104	24 April 2024
***C. albicans* chromosome 1**	5476	5	195	28 May 2024

a Summary of inclusive and exclusive genome sequences used for TAPPR assay design and evaluation. Sequence data were obtained from NCBI Datasets or NCBI Virus, with the number of genomes and the date of retrieval recorded for each target pathogen. The dataset breadth reflects the genetic diversity considered in the design process. No exclusivity analysis was performed for SARS-CoV-2.

To evaluate the reliability of our method for conservation estimation in the performance of assays into the future, we collected complete sequences from NCBI Virus for both SARS-CoV-2 and Mpox for the dates following our collection dates specified in Table 1 and performed in silico inclusivity analysis on the TAPPR designed assays that were synthesized and tested. The datasets used in this future-forward evaluation are summarized in Table 2.

**Table 2:** Sequences for evaluation of future performance.^a^

Target	Taxid	No. of sequences	Dates covered
**Mpox (both Clade I and Clade II)**	10244	1501	16 June 2023–30 June 2026
**SARS-CoV-2**	2697049	176 043	27 July 2022–30 June 2026

a Summary of inclusive genome sequences used for *in silico* estimation of future performance on TAPPR designed assays.

### 2.2 Pipeline

The objective in the development of TAPPR (Fig. 1) was to design a data-centric approach to molecular assay design. Therefore, scalability was the primary focus. The multiple sequence alignment step of traditional molecular assay design approaches provides a significant computational bottleneck, so we use alignment-free methods to overcome this. This allows the design stage to use inputs at the whole-genome range in terms of breadth, and thousands of genome examples in terms of depth. As a side effect, when dealing with complete genome inputs, this alignment-free approach also overcomes issues with genomic rearrangements or differences in convention for start coordinate index in circular genomes. Since the alignment-free approach also means that genomic context is lost when performing set operations, oligos designed using these estimated regions of conservation are not required to be conserved in those same, relative positions across the inclusive sequences. Using an alignment-free approach confers other advantages as well when compared to multiple sequence alignment. MSAs assume that homologous positions have corresponding homologous coordinates, but the coordinates from MSAs are arbitrary and do not have context with any one reference genome. Furthermore, factors like genomic rearrangement means loss of genomic context can be challenging for any downstream analysis. As a result, the pipeline consists of two major components: *design* and *evaluation*. Candidate assays are designed using the fast alignment-free method and then verified for success based on the evaluation tools.

**Figure 1:**
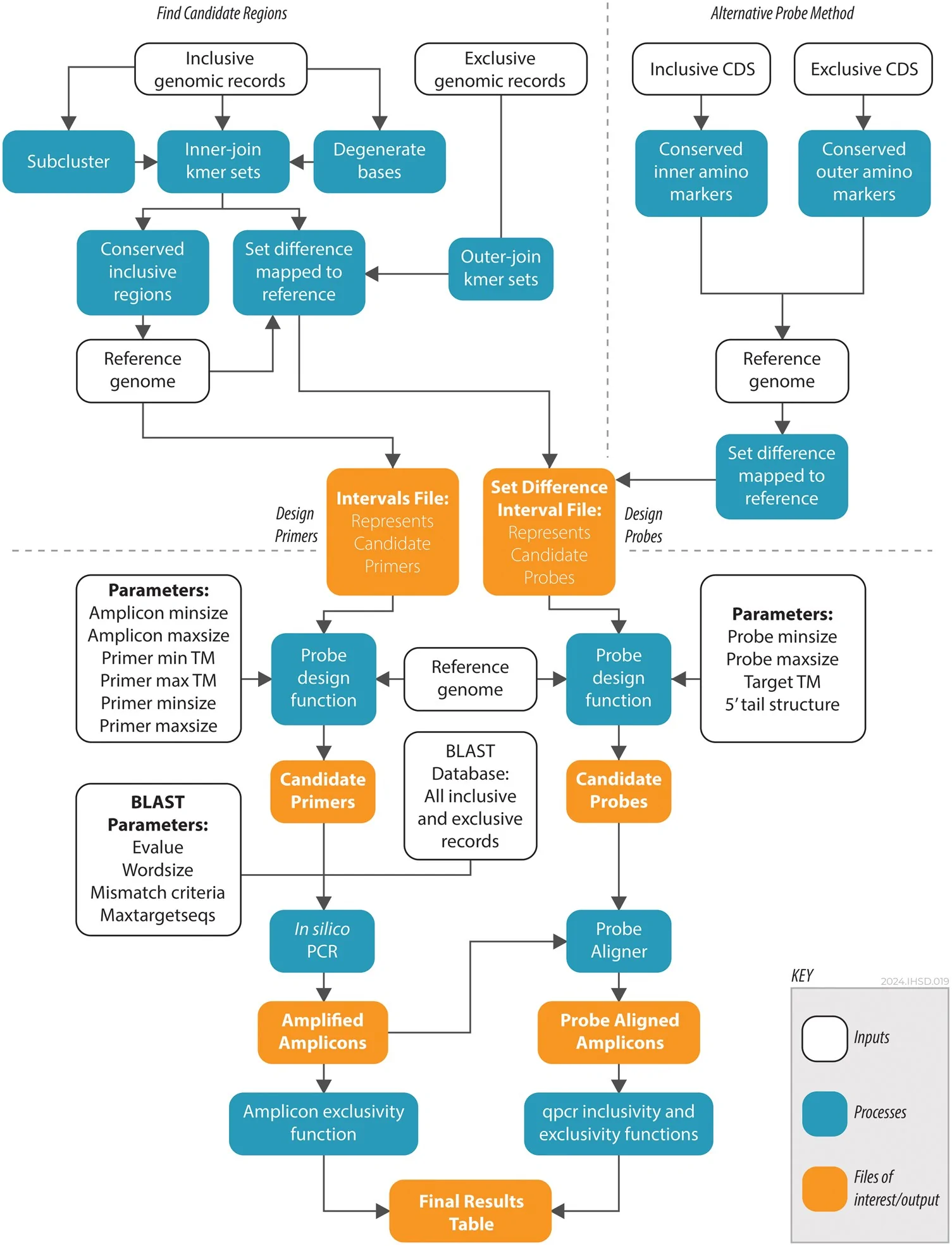
Overview of the TAPPR pipeline for molecular assay design. The workflow consists of multiple stages, including sequence data retrieval, k-mer-based conservation analysis, hierarchical subclustering, marker selection, primer and probe design, and *in silico* evaluation. The pipeline eliminates the computational bottlenecks associated with traditional alignment-based approaches, allowing for scalable and rapid assay development.

### 2.3 Design

TAPPR supports three types of molecular assays which use different reporting systems for detection. This includes SYBR green (where detection is based on amplification of the target), probe-based PCR (where probe hybridization is required for detection), or amplicon sequencing (where the internal sequence identity of the amplicon provides the detection signal). To execute the TAPPR pipeline, the end user must specify requisite design constraints for amplification product sizes and melting temperatures for oligos. Additional optional constraints can be specified, such as the restriction of oligo selection to coding regions. The minimum required input is an inclusive group of genomic records, but assays can be designed and evaluated for exclusivity to another group of off targets if desired. In the SYBR green case and the amplicon sequencing case, only pairs of primers are created. In the probe-based PCR case, an internal oligo is selected as well. All three TAPPR design cases follow a similar workflow, with only small deviations. The pipeline can be run to design and evaluate assays or to evaluate designs from another source.

All TAPPR processes begin by finding regions of conservation among the in-group. These regions of conservation are estimated through iterative inner-joins of k-mer sets across all records of the in-group. This is accomplished using the *kmercountinner.py* script where the main parameter is the choice of k-mer size. A larger k-mer size allows for more specificity in region curation upfront, with the tradeoff of a longer time to answer as the number of canonical k-mers grows exponentially with k-size. A k size of 18 was chosen as a default for balancing time to answer and specificity. Optionally, a reference genome or a specification of an allowed percent variance of genome length can be specified as a way to QC genome lengths. The remaining inner set of k-mers are then assigned back to their originating coordinates in a selected reference genome and merged into larger contiguous regions. By this method, TAPPR avoids any polynomial-time alignment algorithms during the oligo design process. For most closely related, DNA-based targets, this should be sufficient to identify conserved regions for primer picking depending on reaction criteria. In the case that there are not conserved k-mers across the in group due to diversity across the in-group, sub-clustering is used to find a panel that can piece-wise cover the in-group. To achieve this, TAPPR has a hierarchical clustering utility that uses *sourmash* (Irber *et al*. 2024) sketches for quick sequence similarity comparisons and allows for tuning criteria such as the maximum distance in the clustering tree cut or the maximum number of clusters to be generated. For the *clustering.py* step, two criteria can be applied. Clusters can be determined by a maximum number of clusters (*maxclust*) or a maximum cutoff distance (*maxdist*) between clusters. Using *maxclust* can be effective if the user has an *a priori* estimate of how many clusters they expect to emerge, such as along species or clade designations. The k-mer counting method can then be run on each cluster output, and the process can be repeated until non-empty sets of conserved k-mers covering each subcluster are found. The result of this step in the pipeline is a set of conserved primer regions.

To generate candidate primers, the TAPPR pipeline first searches for candidate amplicon regions. To focus on regions likely to produce specific assays to the target of interest and to reduce the search space, TAPPR requires marker regions to be specified. These marker regions are used to design either probe oligos or, in the case of amplification-based specificity, to design primers around. These marker regions can be established through one of a few methods implemented in TAPPR. The first method is the use of k-mer set operations to take the union of all k-mers observed in the exclusive sequences and subtract them from conserved k-mers derived from the inclusive sequences. TAPPR has utilities to perform these set operations on nucleic acid k-mers directly, through *kmercountouter_set.py* and *kmer_set_difference.py.* Like *kmercountinner.py*, the main tuning parameter is choosing a kmer size which should be the same size as chosen for inner kmer counting. However, amino acid k-mers derived from coding domain sequence data from both the inclusive and exclusive groups can also be used. Amino markers unique to the in group are then aligned back to the reference genome using *tblastn* in order to establish a common coordinate system for the primer regions and the marker regions. Like other kmer counting methods in the pipeline, the kmer size is the main tunable parameter but this time the k size is amino acids instead of nucleotides. A kmer size of 8 is chosen as a default for the tradeoff between speed and specificity. In the instances where exclusive data is difficult to define, another method is to simply take the complement of the conserved primer regions file but should be used with caution outside of smaller design cases as the candidate amplicon search space can inflate quickly.

Marker regions are then passed to the probe selection script. Probes are selected along several specifiable criteria, the first of which is a specified maximum and minimum internal oligo size. An optimal melting temperature is also specifiable, which is the highest priority of the design process. A required nucleotide sequence for the end of the 5’ of the design can also be specified. The selection of these parameters will depend on what the desired reaction thermodynamics are. Generally, the probe melting temperature should be higher than the primer melting temperatures. Tuning the allowable minimum and maximum length will have an implicit effect on melting temperatures as longer oligos have higher melting temperatures. The choice of the 5prime HNNNN sequence is a standard practice for maintaining probe stability (Untergasser *et al*. 2012). If possible, each marker region has a single selected best internal oligo returned from this process that can be fed alongside primer regions into the next step, primer selection. The primer selection step considers similar criteria, with requirements for minimum and maximum primer length, minimum and maximum primer temperature, and the minimum and maximum length an amplicon produced by two primers should be. In the case where a probe is designed, primers are selected such that they are around a conserved marker region. If no probe is being designed, such as in a qPCR assay using an intercalating dye as the reporter, the input marker regions can be used to select primers from, to achieve selective amplification. In both primer and probe design, TAPPR considers hairpin, GC content, self-dimerization, cross-dimerization, consecutive character, and consecutive repeated characters through a series of Boolean screening processes for each candidate. The output is a set of primer candidates, which can then be evaluated for performance in the TAPPR evaluation suite.

To evaluate the performance of the candidate assays produced in the design phase, TAPPR performs an *in silico* estimate of amplification products through a python re-implementation of *simulate_PCR* (Gardner and Slezak 2014). For the given primer sets and reaction parameters, such as limits of product sizes and tolerances for primer mismatches to be considered a positive hit, this tool will output a table of predicted amplification products for a specified BLAST database of sequences. For inclusivity analysis, this BLAST database would be composed of inclusive sequence records, and for exclusivity analysis this BLAST database could be composed of the specified exclusive sequences, or another BLAST database such as the NCBI NT database. Other than the curation of simulate PCR database, other specifiable parameters include BLAST parameters for *e-value*, *word_size*, and *max_target_seqs*. The number of allowed mismatches for a sequence to be considered a hit can also be specified, with three as the default. Some number of mismatches are acceptable in the reaction and this can be tuned depending on what is known about the desired PCR mechanics. Minimum and maximum amplicon size can also be specified, where the lower limit of amplicons are bounded by the sizes of the primers (and optionally probe) while the maximum limit is typically bounded by reaction properties. Multiplex evaluations can be turned on using the associated flag as well. If the assay uses a probe reporter, then probe sequences are aligned to the predicted amplicons in an additional step, *probe_alignment.py*, using a mismatch tolerance argument to estimate the success of probe hybridization. Like *simulate_pcr.py* beforehand, probe mismatches with a default of three base pairs can be tuned to the desired number of allowable mismatches to consider a hit. Therefore, inclusivity and exclusivity are conditioned on selection of this cutoff criteria and are left to the end user to decide based on other reaction parameters that may influence the specificity of oligo hybridization.

The size of the outputs from the design tools can vary drastically, depending on the level of conservation in the inclusive and exclusive groups, and leads to the main potential bottleneck of the workflow in the evaluation stage. One solution to achieve quicker time to answer is to randomly subsample an output of thousands of candidates to a few dozen. This may miss the most optimal solution in favor of a functional solution, which is up to the user. For example, in the case of Mpox probe alignment, which is the single longest step in assay generation, one solution may have been to subsample the number of probes considered during the probe alignment step. A future improvement to TAPPR may involve an option to iteratively randomly sub-sample primer and probe candidates for evaluation and stopping early when an acceptable solution is found. This approach would also allow for TAPPR to work with a smaller memory footprint during compute.

Finally, designs may be evaluated for their inclusivity and exclusivity to any sets of desired sequences. While inclusivity and exclusivity have been a consideration throughout the design process, this serves as a final validating step that produces easily readable results. If using qpcr, *qpcr_inclusive.py* and *qpcr_exclusive.py* take mismatch specifications and allow for taxonomy or custom label strategies to determine in or out group hits. If amplification is the exclusive metric, then *amplicon_exclusive.py* should be used with similar label specifications. Finally, *primer_summary_table.py* can then accept the outputs from the inclusive and exclusive analysis to create a summarizing table.

### 2.4 Code availability

The full source code for the TAPPR assay design pipeline is publicly available under an open-source license via GitHub at https://github.com/mriglobal/tappr. This repository includes documentation, example datasets, an example workflow, and scripts necessary to reproduce the pipeline’s core functionalities, including k-mer-based conservation analysis, marker region selection, and oligonucleotide design. All analyses described in this study were conducted using version 1.0 of the pipeline (current as of April 2025).

### 2.5 Evaluation

The performance of TAPPR-designed assays was assessed using a combination of in silico PCR simulations and laboratory validation. Computational evaluations were conducted to determine inclusivity and exclusivity based on simulated amplification across both target and non-target genomes. For each designed assay, an indexed BLAST database containing all inclusive and exclusive sequences was generated, allowing for efficient PCR simulations. Parameters such as minimum and maximum amplicon length, mismatch tolerance, and e-value thresholds were set to reflect typical, real-world PCR reaction conditions. These parameters were identical for each design and are listed in Table 3. Probe-based assays underwent an additional alignment step to confirm probe specificity against simulated amplicons prior to evaluation. Inclusivity is reported as the fraction of total target sequences meeting these criteria over the total number of sequences available for evaluation. If any sequences from the exclusivity group have both the primers and probes all aligning to a template sequence within the mismatch criteria then that oligo set is considered non-exclusive. Following *in silico* evaluation, laboratory testing was performed to validate assay specificity and sensitivity. Positive control materials for each target organism were tested alongside exclusivity controls selected based on TAPPR’s computational exclusivity predictions. Real-time PCR (qPCR) and reverse transcription qPCR (RT-qPCR) were conducted using standard cycling parameters, with assay efficiency measured through serial dilutions where applicable.

**Table 3:** Design pipeline parameters.^a^

Design parameter	Tool	Input value
**K-size for k-mer counting**	*kmercountinner.py, kmercountouter_set.py*	18
**Minimum product size**	*make_primers.py*	60
**Maximum product size**	*make_primers.py*	250
**Minimum primer length**	*make_primers.py*	20
**Maximum Primer Length**	*make_primers.py*	30
**Minimum primer Tm**	*make_primers.y*	56°C
**Maximum primer Tm**	*make_primers.py*	64°C
**Minimum probe size**	*make_probes.py*	18
**Maximum probe size**	*make_probes.py*	28
**Optimum probe Tm**	*make_probes.py*	65°C
**Maximum primer mismatches**	*simulate_PCR.py*	3
**Maximum probe mismatches**	*probe_alignment.py*	3

a Parameters used for the design of the assays evaluated for SARS-CoV-2, Mpox, *C. albicans*, and *M. tuberculosis*.

Based on the availability we had for representative organisms in our inventory, we established a testing array for positive material and, in the case of every assay except the SARS-CoV-2 target, presumptive negative material based on TAPPR’s exclusivity analysis. The strain materials used across the testing is as follows:

**SARS-CoV-2 assay**: the positive material used was the BA.5 variant of SARS-CoV-2. No exclusivity materials were tested.

***C. albicans* assay**: the assay was validated using *C. albicans* as the positive target material. To test exclusivity, the assay was challenged against closely related non-target Candida species, including; *C. utilis*, *C. lusitaniae*, *C. glabrata*, *C. krusei*, *C. parapsilosis*, and *C. tropicalis*.

**Mpox assay**: the positive material was Mpox virus Clade 1. Exclusivity controls included other orthopoxviruses to assess specificity; Vaccinia virus (strains MVA and Wyeth), Rabbitpox virus, and Camelpox virus.

***M. tuberculosis* assay**: the assay was validated using *M. tuberculosis* H37rv as the positive material. Exclusivity testing included a wide range of non-target Mycobacterium species to ensure specificity, including; *M. xenopi*, *M. abscessus*, *M. fortuitum*, *M. intracellulare*, *M. lentiflavum*, *M. marinum*, *M. scrofulacium*, *M. smegmatis*, *M. chelonae*, *M. avium*, *M. gordonae*, *M. kansasii*, *M. malmoense*, *M. mucogenicum*, *M. simiae*, and *M. szulgai*.

For comparative assessment, TAPPR-designed assays for Mpox and SARS-CoV-2 were benchmarked against CDC reference assays using the same qPCR conditions, reagents, and instrumentation. The CDC N1 assay for SARS-CoV-2 (Lu *et al.* 2020) and the CDC Mpox assay [National Center for Emerging and Zoonotic Infectious Diseases (US), Division of High-Consequence Pathogens and Pathology, Poxvirus and Rabies Branch 2022] served as gold-standard comparators for evaluating amplification kinetics and cycle threshold (Ct) values. These comparisons provided insights into TAPPR’s assay performance relative to established diagnostics. The combined *in silico* and laboratory evaluations confirmed that TAPPR-designed assays exhibited high specificity and competitive sensitivity, with performance comparable to or exceeding current fielded assays. The exclusivity predictions made by TAPPR’s computational pipeline were validated experimentally, supporting its utility in rapidly designing molecular assays with minimal cross-reactivity.

### 2.6 Nucleic acid extraction

#### 2.6.1 SARS-CoV-2

SARS-CoV-2 Viral nucleic acid was extracted using the Qiagen Viral RNA mini kit. 140 μL SARS-CoV-2 BA.5 suspended in Viral Transport Media (VTM) was combined with 560 μL ethanol, and vortexed to mix. Samples were incubated for 5 min, and the entirety of this solution for each sample were added to QIAamp mini columns and centrifuged at 6000× *g* for 1 min. 500 μL of buffer AW1 was added, and centrifuged at 6000× *g*, then 500 μL of buffer AW2 was added, and centrifuged at 20 000× *g* for 3 min. Samples were then spun at 20 000× *g* for 1 min to dry. Elution was performed using 60 μL of buffer AVE with incubation at room temperature after addition for 1 min prior to centrifugation at 6000× *g* for 1 min.

#### 2.6.2 *Candida* spp.

*Candida* spp. nucleic acids were extracted using the Qiagen DNeasy blood and tissue kit, using the manufacturer’s protocol with an additional bead beating lysis step. All seven *Candida* species were aliquoted into tubes, and cells were harvested by centrifuging for 10 min at 5000× *g*, the supernatants discarded. Cell pellets were resuspended in 180 μL of buffer ATL and mixed by vortexing before 20 μL of proteinase K was added to this mixed solution. The entirety of this solution was added to Powerbead tubes from Qiagen containing approximately 0.1 mL of glass beads, and these tubes were vortexed at max speed for 1 min. 200 μL of water was added to each sample, and samples were centrifuged at 5000× *g* for 5 min. Supernatants were transferred to new tubes and incubated at 56°C for approximately 1 h. Once incubated, 200 μL of buffer AL was added to each sample, mixed by vortexing, and 200 μL of 100% ethanol was added to each sample and mixed. The entirety of the solution was transferred to DNeasy Mini Spin columns and spun at 6000× *g* for 1 min. 500 μL of buffer AW1 was added to each sample and centrifuged at 6000× *g* for 1 min. Then 500 μL of buffer AW2 was added to each sample and centrifuged for 3 min at 20 000× *g*. 100 μL of buffer AE was added directly to the membranes to elute nucleic acid, and samples were incubated at room temperature for 1 min. Samples were centrifuged at 6000× *g* for 1 min to elute.

#### 2.6.3 Mpox and other Poxviridae species

Historical stocks were provided for use in all experiments involving Poxviridae species. All Mpox and Poxviridae species were previously extracted (and for applicable organisms, at BSL-3), were sterility verified prior to their use.

#### 2.6.4 *M. tuberculosis*

The non-tuberculosis mycobacterium (NTM) and *M. tuberculosis* H37Rv isolate materials were extracted according to the Deeplex Myc-TB protocol. Nucleic acid extracts were quantified using the Qubit 1X dsDNA High Sensitivity kit. Quantification readings along with genome size data for each strain were used to calculate genomic-equivalents-per-microliter.

#### 2.6.5 qPCR and RT-qPCR

All qPCR was performed on either the Bio-Rad CFX 96 or Opus (Hercules, CA, USA), or the Applied Biosystems Quantstudio 5. All Master mix preparations used the proprietary master mixes Taqman or Taqpath by ThermoFisher (Waltham, MA, USA) at 1X concentration for DNA or RNA targets, respectively. All qPCR primers and probes were generated using TAPPR, except for those generated by the CDC–CDC_N1 for SARS-CoV-219, and CDC_Mpox for Mpox20. Primer concentrations used for all assays were 300 nmol, and probe concentrations for all assays were 200 nmol. All reactions consisted of 5 μL sample adds and 15 μL of master mix in triplicate reactions. PCR amplification is based on the fluorescent emission of molecular probes after annealing/elongation. Primers and probes were synthesized by Integrated DNA Technologies (Coralville, IA, U.S.A). Detection probes consisted of a 5’ reporter molecule (6-carboxyfluorescein) with a 3’ quencher (Iowa Black Hole Quencher 2). Thermal cycling parameters were as follows; for DNA targets—95°C for 2 min, followed by 45 cycles of (i) 95°C for 3 s and (ii) 60°C for 30 s, and for RNA targets—25°C for 2 min, 50°C for 15 min, 95°C for 2 min, followed by 45 cycles of (i) 95°C for 3 s and (ii) 55°C for 30 s. Initial experiments were performed using the run cycling parameters listed above. PCR assay conditions were optimized for the TAPPR SARS-CoV-2 designs using thermal gradients varying the temperature of the annealing/extension step from 54°C to 62°C after initial evaluation using the cycling parameters listed. In applicable experiments where assay efficiency was measured, samples were serial diluted in 10-fold serial dilutions to obtain efficiency metrics.

### 2.7 Existing methods

We attempted to compare TAPPR to similar design tasks that could be composed with existing methods. An existing alignment-free method that uses a similar set-object approach, RUCS (Thomsen *et al*. 2017), was used to attempt designs of probe-based qPCR assays. Using matching parameters for Tm, oligo lengths, and PCR product sizes, we ran the RUCS design pipeline on the same data inputs for *C. albicans*, Mpox, SARS-CoV-2, and *M. tuberculosis* used in our TAPPR designs. Additionally, while traditional multiple-sequence-alignment-based approaches are not scalable to the data inputs used in our study, we attempted an alternative approach that uses ViralMSA (Moshiri 2021) to produce a pseudo-MSA and use that alignment input for DECIPHER, an R package that can be used for the design of group-specific primers. While probe-based PCR assays are not specifically designable by DECIPHER, we used this as a close comparison to the process we demonstrated with TAPPR and input the same design parameters regarding Tm and PCR produce sizes.

Primers and probes designed by these tools had their performance evaluated by the same TAPPR components used for evaluation of TAPPR designed assays. This was composed of (i) *simulate_PCR.py* to estimate PCR products, (ii) *probe_alignment.py* when probes were available, and (iii) *qpcr_exclusive.py* to examine if off-target hits were detected. The RUCS pipeline includes a BLAST-based in silico PCR product prediction step, but we used our evaluation modules to provide a better basis of comparison to the TAPPR method.

We intended to also compare results using another recently published tool for molecular assay design name PUPpy (Ghezzi *et al*. 2024) but were unable to use the software. First, the comparison is imperfect as PUPpy requires coding domain sequences while TAPPR accepts full genome inputs. Second, even when conforming to the PUPpy expected input, the code was unable to run in the command line and GUI forms. When using the group primers mode, the program throws a “ResultDB not defined” error, and when not using the group primers mode UniqueGenesList is not defined from what appears to be an issue with the variables being referenced before they are instantiated. While the tool could possibly be fixed, we were unable to get results from the current code repository.

## 3 Results

### 3.1 Computational

All TAPPR assays were designed on a compute node with 64 cores and 1.5 TB of memory, and time-to-answer is provided in Fig. 2 for each process in the pipeline. Specific commands and parameters are outlined in the Supplementary Timing Notes. Maximal multithreading (threads = 64) was used wherever applicable and is the default of TAPPR. In the event of limited RAM, memory chunking modes can be used on applicable scripts. Exact commands run can be found in the Supplementary File  *commands.txt*.

**Figure 2:**
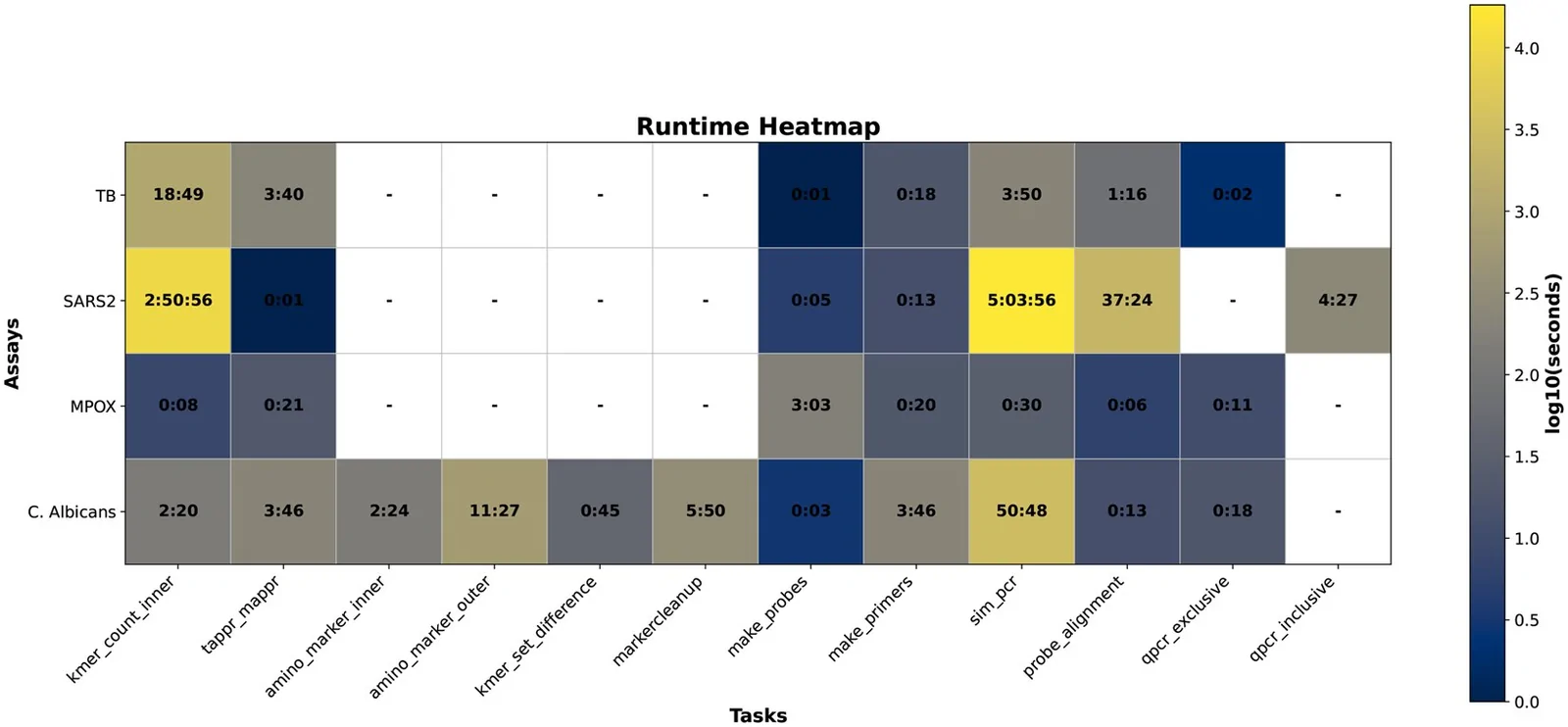
Heatmap showing processing time (in seconds, per color scale) for each stage of the TAPPR pipeline across four assay targets (*M. tuberculosis*, *C. albicans*, Mpox, SARS-CoV-2). Blank boxes denote skipped steps with zero runtime. SARS-CoV-2 exclusivity was assumed due to RNA virus diversity. For *M. tuberculosis*, Mpox, and *C. albicans*, inclusivity was inherent to the inner k-mer set; SARS-CoV-2 required subsampling. Amino marker steps were only used for *C. albicans* due to genome complexity. The heatmap highlights bottlenecks in in-group/out-group comparisons, guiding future scalability improvements.

While existing state of the art primer tools perform similar steps to different components of TAPPR, the novel combination of primer design ideas makes it difficult to profile TAPPR against other software. Primer3 is a prolific tool by itself and the back end of many other analyses, but it does not perform region curation to identify regions of exclusivity like TAPPR does. Tools like DPPrimer (Gahoi *et al*. 2013) are able to curate regions before primer design, but the inclusion of a multiple sequence alignment step limits the corpus of genomic records that may be used as an input, in terms of both genome size and the number of genomes. As a comparison for the primer design component, we compare TAPPR’s primer selection step to Primer3 using the same input regions. As a comparison for the region curation component, we compare TAPPR’s region curation to a multiple sequence alignment for a similar sized input. In comparison to Primer3 design for the same input regions as TAPPR, we find no agreement between our presented guides and the Primer3 designs. The Primer3 designs can be found in the Design Table 1, available as supplementary data at *Bioinformatics Advances* online. In comparison to multiple sequence alignment, we run the *M. tuberculosis* example through the MAFFT (Katoh *et al*. 2002) multiple sequence aligner. While TAPPR was able to find conservation for the 528 bacterial genomes in 18 min and 49 s, the multiple sequence alignment ran for two weeks before termination on comparable hardware.

Evaluation of the SARS-CoV-2 lineage-stratified subsampling approach showed that the resulting assays designed in each of the 10 iterations were invariant to *in silico* inclusivity analysis on the complete SARS-CoV-2 dataset from Table 1. The mean inclusivity of the designed assays was 99.9999% ± 0.0001%.

Analysis on designed assay for SARS-CoV-2 and Mpox on new sequences published after the data cutoff also showed high inclusivity performance was retained suggesting the alignment-free method for estimation of conservation was a good predictor of future conservation from the respective taxonomic groups. The *in silico* inclusivity performance on the datasets from Table 2 using the assay designs described in Table 5 are summarized in Table 4.

**Table 4:** Inclusivity performance on future data.^a^

Target	Assay ID	Inclusivity coverage
**SARS-CoV-2**	SARS2_4	0.999943
**SARS-CoV-2**	SARS2_5	0.999813
**SARS-CoV-2**	SARS2_6	0.999926
**Mpox**	Mpox_1019	1.0
**Mpox**	Mpox_249	1.0
**Mpox**	Mpox_389	1.0

a In silico inclusivity analysis of the synthesized assays on complete genome sequences for SARS-CoV-2 and Mpox that were submitted to NCBI after the cutoff date for sequences used in the design of the assays.

**Table 5:** Oligos synthesized and tested.^a^

Oligo ID	Sequence (5’ to 3’)
**SARS-CoV-2**	
** SARS2_4_Forward**	TGTGATAGAGCCATGCCTAA
** SARS2_4_Reverse**	CCACCTGGTTTAACATATAGTGAA
** SARS2_4_FAM**	TTGAGTGAAATGGTCATGTGTGGCG
** SARS2_5_Forward**	GTAATGCACATGTAGCTAGTTGTGAT
** SARS2_5_Reverse**	ACACACTTAATAGCTTTAGGGTTACC
** SARS2_5_FAM**	TCGTGGACAGCTAGACACCTAGTCA
** SARS2_6_Forward**	AGACAGTTTCATCAAAAATTATTGAAATC
** SARS2_6_Reverse**	TTAGGCATGGCTCTATCACA
** SARS2_6_FAM**	ATAGCCGCCACTAGAGGAGCTACT
** CDC_N1_Forward**	GACCCCAAAATCAGCGAAAT
** CDC_N1_Reverse**	TCTGGTTACTGCCAGTTGAATCTG
** CDC_N1_Fam**	ACCCCGCATTACGTTTGGTGGACC
**Mpox**	
** Mpox_1019_Forward**	TAGAAGAGCGAATATCTCGTCTGAC
** Mpox_1019_Reverse**	TTTTAATAATGGTGTTGATGTGGAGTCTA
** Mpox_1019_FAM**	TGACTTATGCTGCTGAACTACATCTCA
** Mpox_249_Forward**	AGCACCATCTATTCCAAATAATCAGAAA
** Mpox_249_Reverse**	ATGTAAAGCAGCGAGCATATATCATC
** Mpox_249_FAM**	TGGCAAAGCATCACAGAATCCGAGTA
** Mpox_389_Forward**	CTCAATCTTGTTATCGGGATCCAATT
** Mpox_389_Reverse**	AACGGACTAAGCTCATGTCCAA
** Mpox_389_FAM**	CCATTCACGATCGAGGGTTTAATGACA
** CDC_Mpox_Forward**	GGAAAATGTAAAGACAACGAATACAG
** CDC_Mpox_Reverse**	GCTATCACATAATCTGGAAGCGTA
** CDC_Mpox_FAM**	AAGCCGTAATCTAGTTGTCTATCGTGTCC
***C. albicans***	
** calbicans_1120_Forward**	TATTCAGACTGGTACAAATGGTTTGG
** calbicans_1120_Reverse**	TATGCAGATTATTTCGTGGTTGTCTC
** calbicans_1120_FAM**	ACCAACGGCGACAATGATGGGA
** calbicans_207_Forward**	TATTGCACACACTAATATGGGACAAC
** calbicans_207_Reverse**	ATCAGAGACTAGACATGAAAGTCCC
** calbicans_207_FAM**	ACCCATCACGGAAATCGTGGGAG
** calbicans_213_Forward**	GTTGGCTTCATTACCGTATTAGCT
** calbicans_213_Reverse**	GTACATATGAAAATCAAGGACGTGGT
** calbicans_213_FAM**	TCGATCACAGCTGTTCCTTGGCA
***M. tuberculosis***	
** H37rv_Forward**	ACTTAACGCTTCGTTGACCAAAG
** H37rv_Reverse**	TGCAGACCAGTACGAGTTCAC
** H37rv_FAM**	TCAGACAACGGAATGGCCTAGCC

a Down-selected TAPPR-designed assays tested for laboratory validation, along with comparator assays from the CDC, when available. TAPPR-designed assays are those without the CDC prefix in the Oligo ID. The tested assays include those targeting Mpox, SARS-CoV-2, *M. tuberculosis*, and *C. albicans*, representing a range of viral, bacterial, and fungal pathogens.

### 3.2 Laboratory validation

The TAPPR pipeline generated molecular assays for both bacterial and viral targets (Table 5), with laboratory validation demonstrating specificity and amplification efficiency. For *M. tuberculosis* and *C. albicans*, exclusivity testing showed that the assays amplified their intended targets while avoiding cross-reactivity with closely related species (Fig. 3). The ability to distinguish between target and non-target genomes suggests that TAPPR’s approach to primer and probe selection is effective for generating assays with high specificity. For viral targets, assays designed for Mpox and SARS-CoV-2 were compared to existing CDC assays (Fig. 3). Initial evaluation of the SARS-COV-2 designs showed suboptimal amplification (Fig. 1, available as supplementary data at *Bioinformatics Advances* online). Since reaction parameters for the CDC N1 assay had an extension stage temperature setting of 55°C, while our design criteria for primers were between 56°C and 64°C, we optimized the reaction settings for the best performing assay, SARS2_4, as described in Methods. After optimization of the annealing temperature parameter (see Methods), the SARS-CoV-2 assay produced similar amplification curves to the CDC N1 assay, indicating comparable sensitivity. The Mpox_1019 assay, which also showed equivalent amplification curves to the CDC assay, was selected for comparison as off-target amplification of Modified Vaccinia Ankara (MVA) was observed in two of the other candidate assays, MPox_249 and Mpox_389 (Fig. 2, available as supplementary data at *Bioinformatics Advances* online). The source of the discrepancies between *in silico* estimation and wet lab evaluation was found to be a result of indels in the target primer regions that, despite resulting in more than three mismatches in the primer alignments, still allowed for launch of amplification. Nonetheless, these results show that TAPPR can identify conserved primer regions and suitable targets for diagnostic assay development, even when genetic variation within a target clade presents design challenges.

**Figure 3:**
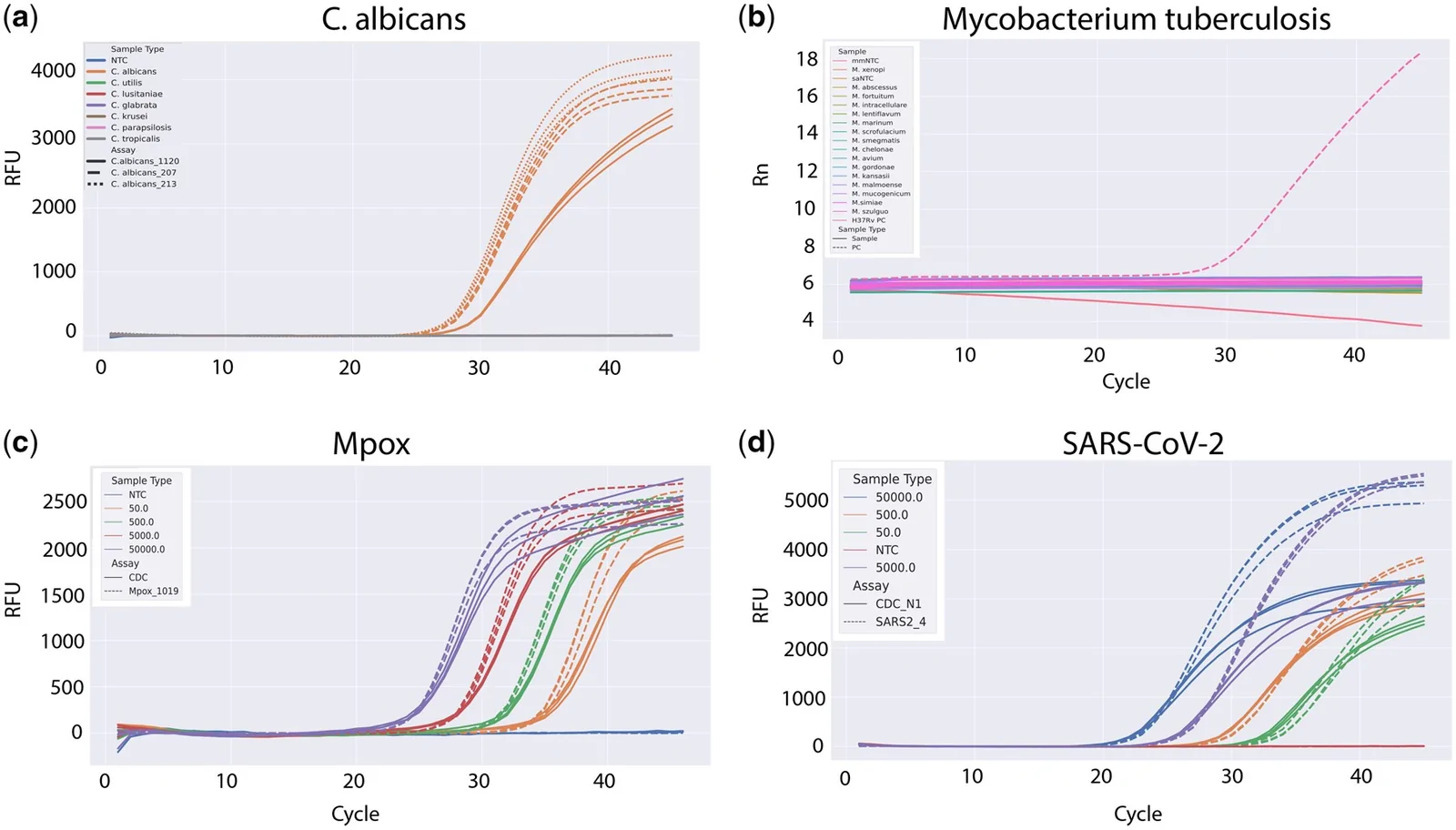
Real-time qPCR amplification plots demonstrating the specificity and competitiveness of TAPPR-designed assays. (a) Exclusivity testing for cellular targets *C. albicans* and (b) *M. tuberuclosis* against closely related near-neighbor species, demonstrating the absence of nonspecific amplification. (c) Competitive evaluation of TAPPR-designed viral assays for Mpox and (d) SARS-CoV-2 against currently fielded CDC assays. The dotted line represents TAPPR-designed assays, while the solid line represents CDC assays, indication similar or superior performance of TAPPR assays across Ct values.

A computational assessment of the TAPPR workflow showed variation in processing time across different pathogens, with certain steps—such as k-mer filtering and exclusivity analysis—requiring more resources depending on genome complexity (Fig. 2). In cases where specific pipeline steps were unnecessary for a given target, those processes were skipped, reducing overall runtime. The heatmap visualization of processing time across different stages of the workflow highlights areas where further optimization could improve efficiency. These findings demonstrate that TAPPR is capable of designing molecular assays that perform well in laboratory validation while maintaining reasonable computational efficiency. The combination of empirical validation and pipeline performance analysis suggests that TAPPR could be a useful tool for developing diagnostic assays, particularly when large genomic datasets need to be processed efficiently.

### 3.3 Comparison to existing methods

In our attempts to provide comparisons between existing tools we encountered several challenges both in terms of pipelines completing successfully and unsatisfactory outputs with respect to the design task. Despite attempting to overcome certain scaling bottlenecks, such as using ViralMSA to generate MSA objects, scaling for these design methods continued to be an issue. Especially when scaling to over one million SARS-CoV-2 records, both RUCS and DECIPHER struggled to handle this volume of sequence instances with some failures resulting from operating system limitations (such as the case with RUCS where each sequence identifier is expanded into an argument for a system call), or with DECIPHER where the primer design algorithm is not computationally efficient enough to produce output in a reasonable amount of time. A summary of these attempted alternative design approaches is summarized in Table 6.

**Table 6:** Summary of results for alternative design tools.^a^

Method	Target	No. of candidate assays	No. of exclusive assays	Notes
**RUCS**	M. tuberuclosis	0	0	No primers reported
**RUCS**	SARS-CoV-2	N/A	N/A	Pipeline error: argument li
**RUCS**	Mpox	193	0	RUCS assigned assays as exclusive which have 1 mismatch in the interior of the oligos
**RUCS**	*C. albicans*	10 000 (199 subsampled for evaluation)	138	
**DECIPHER**	*M. tuberculosis*	N/A	N/A	Up to max iterations of 20 could not find primers sets to cover 100% of *M. tuberculosis* sequences
**DECIPHER**	SARS-CoV-2	N/A	N/A	Primer design never completed (>20 h)
**DECIPHER**	Mpox	12 575	0	Only 1 primer pair 100% inclusive to Mpox. No primers exclusive to Mpox using TAPPR exclusivity analysis
**DECIPHER**	*C. albicans*	N/A	N/A	Primer design never completed (>20 h)

a Summary of outputs from RUCS and DECIPHER for the *M. tuberculosis*, SARS-CoV-2, Mpox, and *C. albicans* target groups using the same input data use in the TAPPR design pipelines. Exclusivity evaluation was performed using the TAPPR exclusivity tools. In many instances primer design has not completed after >20 h of wall clock time.

In the cases where we were able to obtain candidate designs, the TAPPR evaluation pipeline indicated in several circumstances that candidate assays would not meet the exclusivity criteria that was part of the design task. No pipeline for the prediction of off target effects should be considered authoritative, and there are various levels of complexity that can be introduced for the prediction of PCR amplification efficiency. Several of both RUCS and DECIPHER design primer pairs were found to only have a single mismatch in the forward and reverse primers and probe sequence for off-target template sequences. An example predicted amplicon for Cowpox is shown in Fig. 4. These mismatches were also observed to appear in the interior of the primers, not close to the 3’ ends, and therefore, would not be predicted to have a large effect on amplification efficiency (Bru *et al*. 2008). While the single mismatch in the interior of the probe sequence could have a large effect on signal, it is difficult to predict without laboratory testing, but complete elimination of the signal would not be expected (Yao *et al*. 2006). The TAPPR evaluation tools currently use a simple mismatched based criteria for cross-reactivity prediction, but the default parameters used for the designs we present here are much more strict (>3 mismatches). This is compounded by the fact that, while there were multiple candidate assays produced by RUCS for the Mpox target, they all were centered on this single probe sequence, only varying by different combinations of primers. If this particular assay was revealed to be cross-reactive in validation, there are no alternative designs to test from RUCS. This was another distinguishing feature for TAPPR. With the exception of *C. albicans*, the number of candidate assays output by the TAPPR pipeline exceeded those produced by the two alternatives we examined.

**Figure 4:**
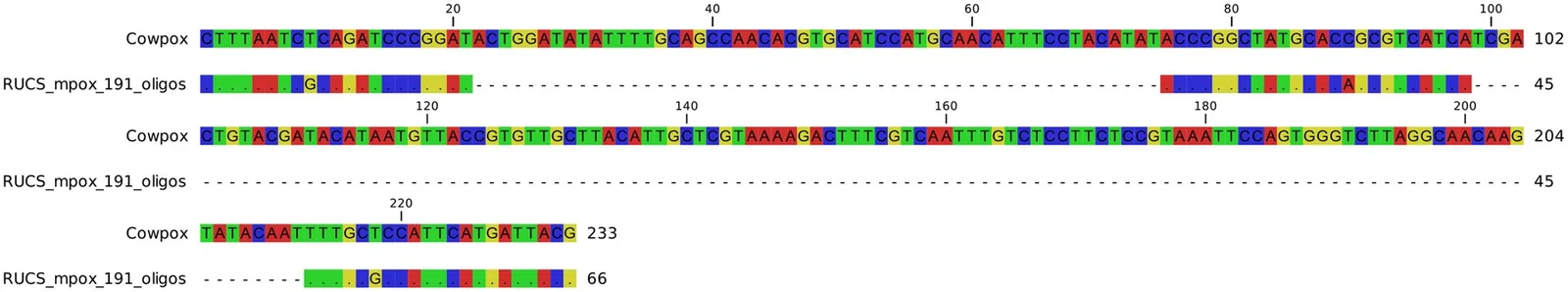
Alignment of the RUCS mpox_191 forward and reverse primers and probe sequences to a Cowpox amplicon predicted by the TAPPR evaluation pipeline. Perfectly matching bases are indicated by dots while mismatches are indicated by the mutated base character.

Finally, scalability issues resulted in failures of these alternative pipelines, both in terms of successful completion of the analysis, but also in the time-to-answer in the instances that they completed. For the RUCS methods that produced any output, the *M. tuberculosis* assay completed in 146 min on the same hardware as our TAPPR evaluation. Mpox took 33 min and *C. albicans* took 246 min. SARS-CoV-2 could not complete due to RUCS’s implementation expanding the input directory of files into a list of inputs the size of the number of files in the directory. This led to 1 011 430 arguments passed to RUCS which well exceeds the operating system argument limit. While RUCS shares a similar, alignment-free algorithmic approach to TAPPR, it suffers from many implementation limitations such as no parallelization and many rate-limiting compute operations written in pure python. For instance, RUCS does not utilize python’s built-in set objects and re-implements a pure python set data structure and methods. The performance cost of this alone, where we expect there to be millions of k-mers contained in set objects for bacterial genomes or larger, is substantial. The RUCS pipeline is also no longer supported as of September 2020.

## 4 Discussion

The TAPPR pipeline demonstrates significant potential for improving molecular assay design, particularly in its adaptability and scalability across diverse pathogens. However, the results of this study also highlight several areas for refinement and opportunities for future enhancements.

One key insight from the Mpox assay validation is the reliance on intergenic regions for target selection. While these regions offer conserved sequences suitable for amplification, they are also prone to structural variations such as insertions and deletions, which can compromise assay specificity. This was evident in two assays that exhibited off-target detections with MVA virus due to indels in intergenic regions targeted by the primers. Importantly, we have included options in the TAPPR pipeline that allow designs to be constrained to coding regions, where these types of indel events are less likely to occur without deleterious effects. While use of these options can reduce the probability of these events, these off-target detections are ultimately attributed to the way that product estimation is performed *in silico.*

Currently, TAPPR evaluates specificity using mismatch-based heuristics, which, while often effective, do not fully account for the complexities of thermodynamics and secondary structure formation that influence amplification efficiency. To address this limitation, ongoing efforts are focused on integrating advanced predictive models into the pipeline (Knight *et al.* 2025). These enhancements, informed by both computational and empirical studies, aim to provide a more nuanced evaluation of primer and probe performance, ultimately increasing the reliability of assay designs. Despite these challenges, the performance of TAPPR-designed assays was highly encouraging. For instance, the SARS-CoV-2 assays performed comparably to the CDC’s gold-standard nucleocapsid-targeting assays, even without the theoretical copy number advantage of highly transcribed target regions. This result underscores the robustness of TAPPR’s alignment-free approach in identifying conserved regions, enabling high sensitivity and specificity regardless of target abundance. For *M. tuberculosis* complex, the assays successfully targeted a region spanning an intergenic area and a hypothetical protein. This innovative dual-targeting approach highlights TAPPR’s flexibility in addressing diverse genomic architectures, even when traditional conserved targets are unavailable. Moreover, the same techniques can be readily applied to the rapid design of assays for NTM, which present challenges due to their genetic diversity and close phylogenetic relationships with other mycobacteria.

Looking ahead, future development will focus on expanding TAPPR’s capabilities. The first improvement is to include degenerate motif targeting as an alternative approach to sub-clustering of highly variable sequences. By enabling the detection of broader clades or strains with highly variable genomes, this feature will enhance TAPPR’s role in addressing emerging infectious diseases and reduce the need for frequent redesigns as pathogens evolve. While TAPPR’s current approach is to identify, empirically, areas of conservation, another future improvement would be a predictive model for genomic loci that are likely to continue to be conserved in the future as input for assay design.

In summary, the results of this study validate TAPPR’s potential as a transformative tool for molecular assay design and for the continued evaluation of current molecular assays against new data as it becomes available. The pipeline’s ability to overcome traditional computational bottlenecks, combined with its adaptability to diverse pathogens and genomic architectures, positions it as a critical resource for biosurveillance, public health applications and outbreak preparedness. Further enhancements, including predictive modeling and degenerate motif targeting, will continue to refine TAPPR’s utility and expand its impact on infectious disease diagnostics.

## Supplementary Material

vbag274_Supplementary_Data

## Data Availability

The accession numbers for all of the sequence data underlying this article are available in the publication_accessions directory at in the Github repository https://github.com/mriglobal/tappr.
