# Supplementary material for: TAPPR PCR assay design—targeted, automated, primer and probe retrieval for scalable molecular assay design
Source: Bioinform Adv. 2026 Sep 17;6(1):vbag274. doi: 10.1093/bioadv/vbag274 (PMC13630449; doi:10.1093/bioadv/vbag274)
Supplement: Supplementary file 1 [file vbag274_supplementary_data.zip › Supplementary_figure_1_TAPPR.pdf]

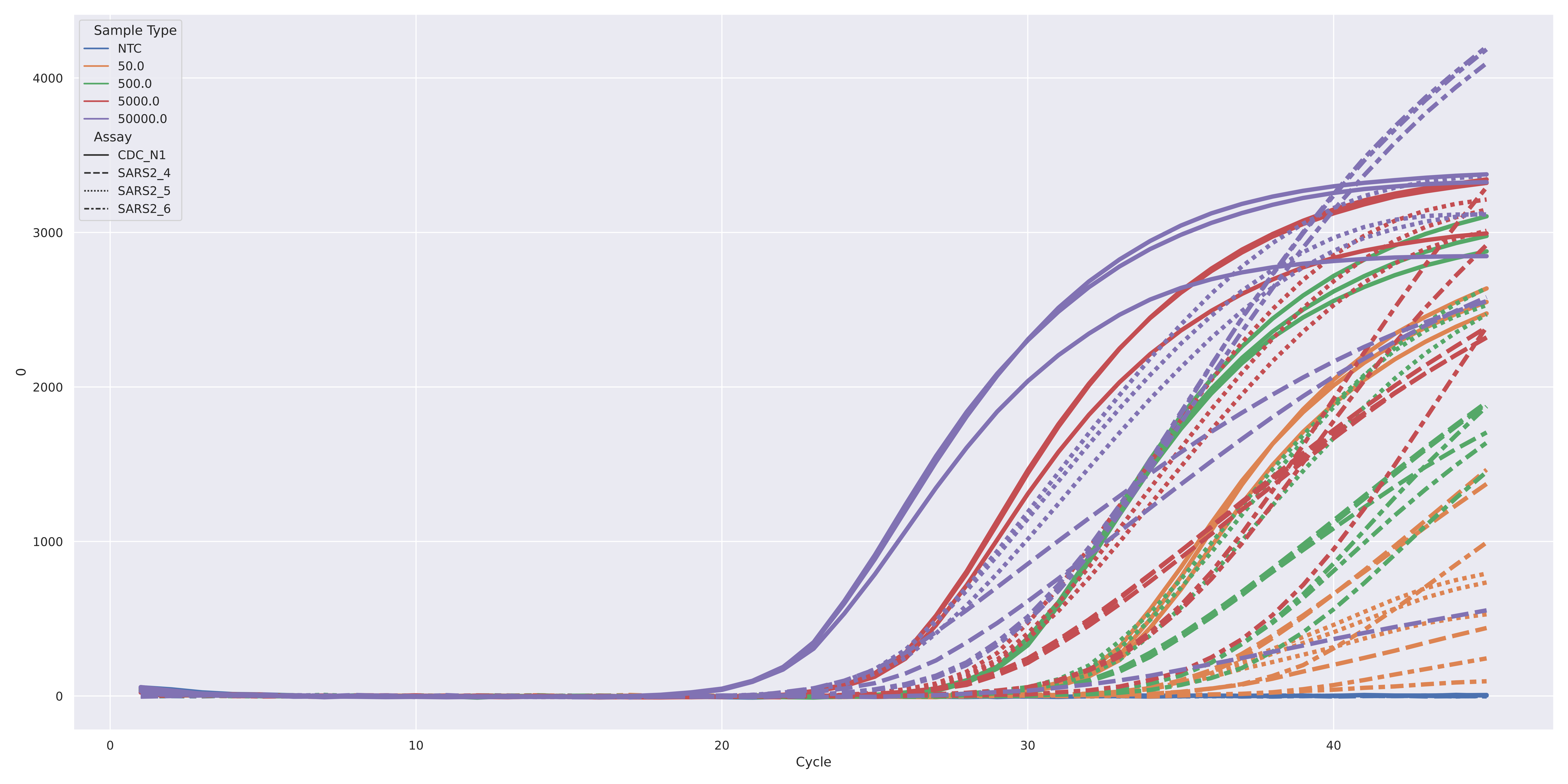
Sample Type
NTC
50.0
500.0
5000.0
50000.0
Assay
CDC_N1
SARS2_4
SARS2_5
SARS2_6
0
1000
2000
3000
4000
0
10
20
30
40
Cycle
0
