# Supplementary material for: TAPPR PCR assay design—targeted, automated, primer and probe retrieval for scalable molecular assay design
Source: Bioinform Adv. 2026 Sep 17;6(1):vbag274. doi: 10.1093/bioadv/vbag274 (PMC13630449; doi:10.1093/bioadv/vbag274)
Supplement: Supplementary file 1 [file vbag274_supplementary_data.zip › Supplementary_figure_2_TAPPR.pdf]

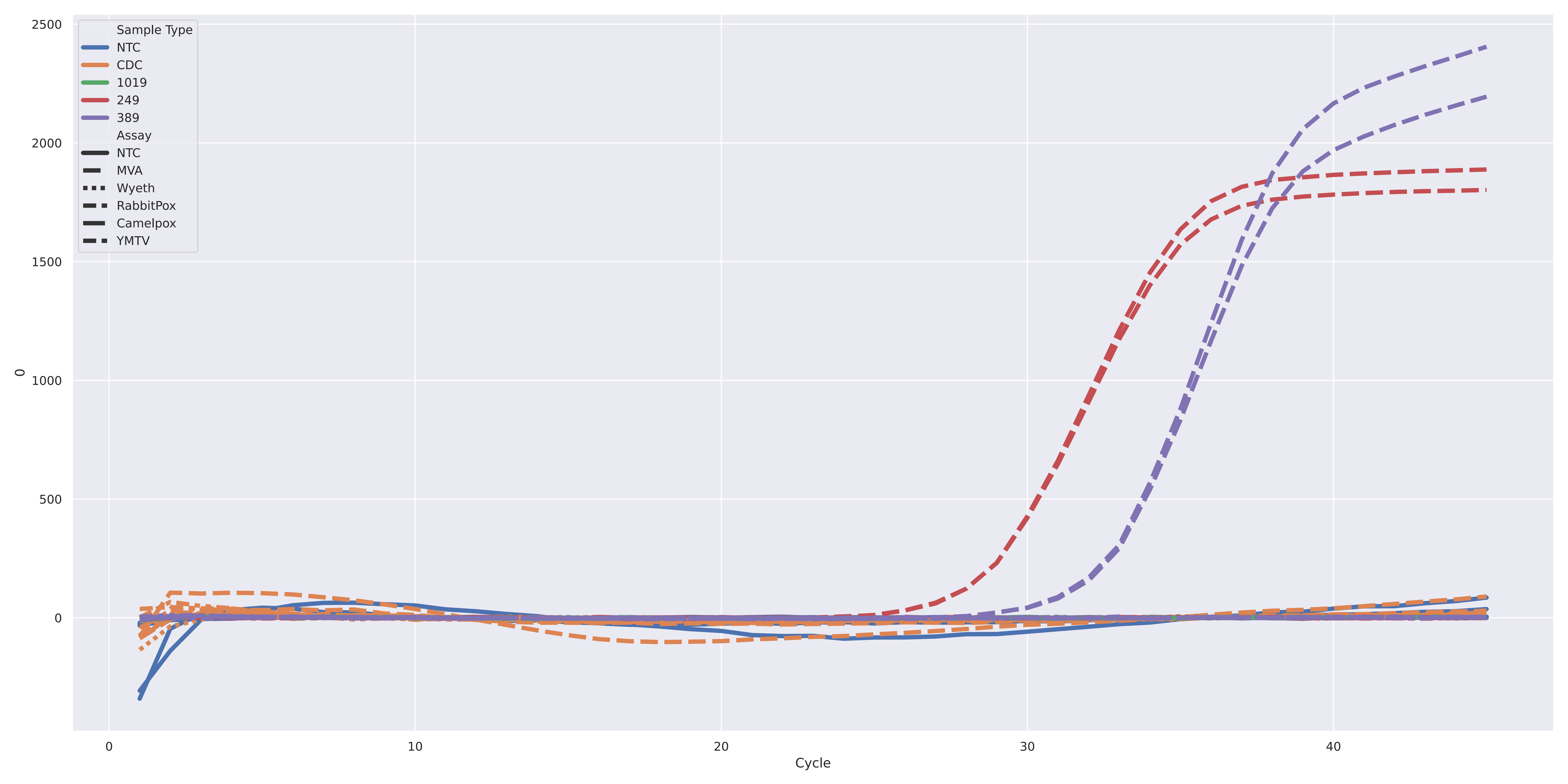
Sample Type
NTC
CDC
1019
249
389
Assay
NTC
MVA
Wyeth
RabbitPox
Camelpox
YMTV
2500
2000
1500
1000
500
0
0
10
20
30
40
Cycle
